# Aromatic thermosetting copolyester bionanocomposites as reconfigurable bone substitute materials: Interfacial interactions between reinforcement particles and polymer network

**DOI:** 10.1038/s41598-018-33131-5

**Published:** 2018-10-05

**Authors:** Mete Bakir, Jacob L. Meyer, Andre Sutrisno, James Economy, Iwona Jasiuk

**Affiliations:** 1 Department of Mechanical Science and Engineering, University of Illinois at Urbana-Champaign, Urbana, IL 61801 USA; 2 Department of Materials Science and Engineering, University of Illinois at Urbana-Champaign, Urbana, IL 61801 USA; 3 NMR/EPR Laboratory, School of Chemical Sciences, University of Illinois at Urbana-Champaign, Urbana, IL 61801 USA; 4 ATSP Innovations, Champaign, IL 61820 USA

## Abstract

Development of porous materials consisting of polymer host matrix enriched with bioactive ceramic particles that can initiate the reproduction of cellular organisms while maintaining *in vivo* mechanical reliability is a long-standing challenge for synthetic bone substitutes. We present hydroxyapatite (HA) reinforced aromatic thermosetting copolyester (ATSP) matrix bionanocomposite as a potential reconfigurable bone replacement material. The nanocomposite is fabricated by solid-state mixing a matching set of precursor oligomers with biocompatible pristine HA particles. During endothermic condensation polymerization reaction, the constituent oligomers form a mechanochemically robust crosslinked aromatic backbone while incorporating the HAs into a self-generated cellular structure. The morphological analysis demonstrates near-homogenous distributions of the pristine HAs within the matrix. The HAs behave as a crack-arrester which promotes a more deformation-tolerant formation with relatively enhanced material toughness. Chain relaxation dynamics of the nanocomposite matrix during glass transition is modified via HA-induced segmental immobilization. Chemical characterization of the polymer backbone composition reveals the presence of a hydrogen-advanced covalent interfacial coupling mechanism between the HAs and ATSP matrix. This report lays the groundwork for further studies on aromatic thermosetting copolyester matrix bionanocomposites which may find applications in various artificial bone needs.

## Introduction

Synthetic bone substitution is the second most frequent transplantation procedure, following blood transfusion, with over two-million surgical operations worldwide per year incurring an estimated cost of $2.5 billion^1–3^. In a broad sense, the replacement process seeks to repair bone defects in orthopaedic, neurosurgical, and dental practices in which the bone substitutes induce healing through reconstruction of the bone defects or serve as implants^4–6^. Generally, several types of bone substitutes have been adopted so far including bone grafts and synthetic scaffolds for bone reconstruction^3^. Autologous bone grafting - utilizing a bone substance obtained from a donor site - is still considered the gold standard, while biomaterials or synthetic scaffolds hold promises as alternative materials^7,8^. At present, the state-of-art bone substitutes are rather customized, being more than mere replacement materials, to individually generated composite biomaterials that can host bone-forming cells to allow expedited defect rehabilitations^9^.

More recently, polymer bionanocomposites have drawn attention in the development of synthetic bone substitutes^10^. Polymer bionanocomposites are combinations of an inorganic phase (bioactive reinforcements) and a polymer phase (host matrix), which can serve two primary purposes: improved biomechanical properties and enhanced biological activity for the polymeric domains^11^. Calcium phosphate (CaP) and hydroxyapatite (HA) are the most well-adapted inorganic cements utilized in bone replacement applications, as they are biocompatible, have genuine morphologies akin to mineral phase of bone, and enable protein adhesion to stimulate cell proliferation^12^. Regarding the polymeric matrix of the nanocomposite replacements, prominent polymers are poly(lactic acid), poly(glycolic acid), and poly(*ε*-caprolactone), which undergo a diffusion-driven hydrolytic decomposition process in *in vivo* conditions^13^. These polymers, as well as their derivatives, are subjected to bulk erosion which allows for new bone to form and replace them. Regarding the enhancement of interfacial interactions between the nanofiller reinforcements and polymers matrices, the most viable approach is to utilize selectively surface functionalized nanofiller particles through an *in situ* polymerization process^14,15^. Via this approach, the nanofiller particles conjugate with polymer chains and are immobilized within the matrix. This method also enables a uniform dispersion of nanofiller particles within polymer matrix^16,17^. Hence, artificial bone scaffold nanocomposites demonstrate improved mechanical strength and enriched biocompatibility. Along with this line, the permanent implant applications require alternative polymer systems that ensure mechanochemical reliability in *in vivo* as well as forming a robust interfacial adhesion mechanism with the bioactive particles for ultimately improved structural properties.

Aromatic thermosetting copolyester (ATSP), introduced in the late 1990s, utilizes economical, conveniently processable and highly cross-linkable oligomers to form a high-performance polymer system^18–20^. The crosslinked network of the ATSP morphology is composed of an aromatic polyester backbone interconnected via covalent single/double oxygen bonds which enables superior physical properties and provides outstanding chemical inertness^21,22^. Besides, ATSP is conveniently reconfigurable into different physical forms including foam, fully-densified bulk part, coating, and adhesive, which therefore can address various synthetic bone applications^16,17,19,20^. More recently, ATSP nanocomposites have been developed in part by adapting a facile production route via solid-state mixing of precursor oligomers with nanofiller particles. As well, the ATSP nanocomposites uniquely enable the near-homogenous distribution and functional incorporation of nanofillers which then constitute significantly enhanced physical properties^23^. Earlier work on ATSP shows direct-contact cytotoxicity test results that fibroblasts remain healthy and still adhered to the ATSP specimen following an incubation period^24^. Hence, the ATSP matrix enriched with bioactive reinforcements can be a promising bionanocomposite candidate as an insoluble artificial bone substitute material due in part to water and salt aging resistance^25^. For example, owing to the strong adhesive bonding formed with most metals including titanium, significant specific impact energy absorption capacity, and promising tribological properties of low coefficient of friction, superior wear, and abrasion resistances, ATSP bionanocomposites are strong candidates for orthopaedic implant applications^19,20,24–26^.

In this study, we demonstrate the hydroxyapatite bioceramic particles reinforced aromatic thermosetting copolyester matrix bionanocomposite. The scope of this paper is reserved to give an insight into interfacial interaction and adhesion mechanisms between the HAs and ATSP matrix, which may lead to systematical biocompatibility analysis of the ATSP bionanocomposites in a following work. Herein, we initially investigate the physical-chemical effects of the HAs on the thermal polymerization reaction. To understand the structural state of the HAs in the ATSP matrix, morphological analysis is performed on the nanocomposite. Mechanical characterization reveals a crack-arresting mechanism induced by the HAs that considerably enhances material deformation tolerance. Besides, glass transition characteristics are altered by segmental confinement of the polymer network instigated by the HAs. Chemical spectroscopy of the backbone chain configuration shows the advancement of a hydrogen-driven covalent interfacial attachment between the HAs and ATSP matrix.

## Results

### Solid-state mixing of precursor oligomers and hydroxyapatite particles

**Figure 1:**
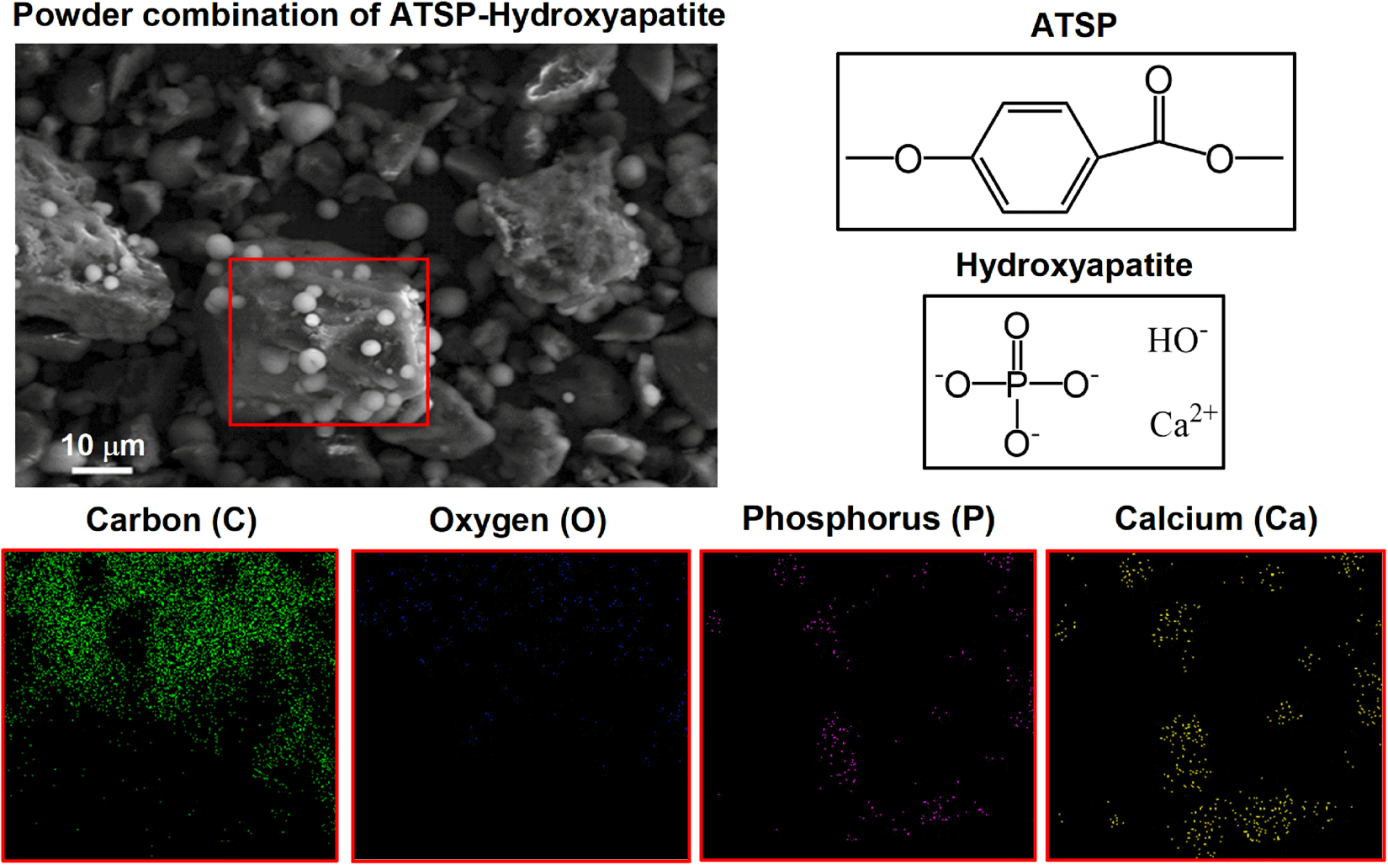
Microstructural analysis of solid-state mixed precursor oligomers and hydroxyapatite particles. Scanning electron microscopy image of uncured powder combination of an precursor oligomer particle (appears darker) decorated with hydroxyapatite particles (appears brighter). Energy dispersive X-ray spectroscopy surface maps of elemental composition (C, O, P, and Ca) of the combination highlighted in the red frame. Chemical representations of the ATSP backbone chain and hydroxyapatite structures.

First, we discuss the effects of the solid-state mixing process on the distribution of the HA particles within the oligomer powder domain. A 10 wt% of the HA particles is incorporated with the precursor carboxylic acid and acetoxy oligomer groups (premixed at 1:1 wt ratio) which forms the precuring ATSP-HA combination, which is then subjected to a thermal polymerization process. Hence, the ATSP-HA nanocomposite is obtained through an *in situ* polycondensation reaction between the functionalized constituent precursor oligomers while the HA particles are also present^17,19^ (refer to Materials and Methods section for details). The polymerization process generates a crosslinked polymer network while releasing acetic acid as a reaction by-product. The acetic acid evolves in the gas form at ~200 °C during the reaction, which acts as a blowing agent to develop a porous matrix morphology. In Fig. 1, a Scanning Electron Microscope (SEM) image demonstrates HA particles (white spheres) effectively dispersed over an oligomer particle. We highlight that, due to the presence of the interparticle van der Waals forces, the HAs are pinned down on the surface of the oligomer particles. The representative chemical configurations given in the same figure show that the constituent oligomer chains, as well as the ATSP backbone chain, comprise H, C, and O while the HA contains H, O, P, and Ca. Through a correlative analysis using an Energy-Dispersive Spectrometer (EDS), elemental mapping of the very same region reveals the presence of the corresponding elements. The content of C dominates over other elements due to the abundance of C in the precursors. More importantly, P and Ca element maps unveil the HA particles in the domain.

### Characterization of polymerization reaction

**Figure 2:**
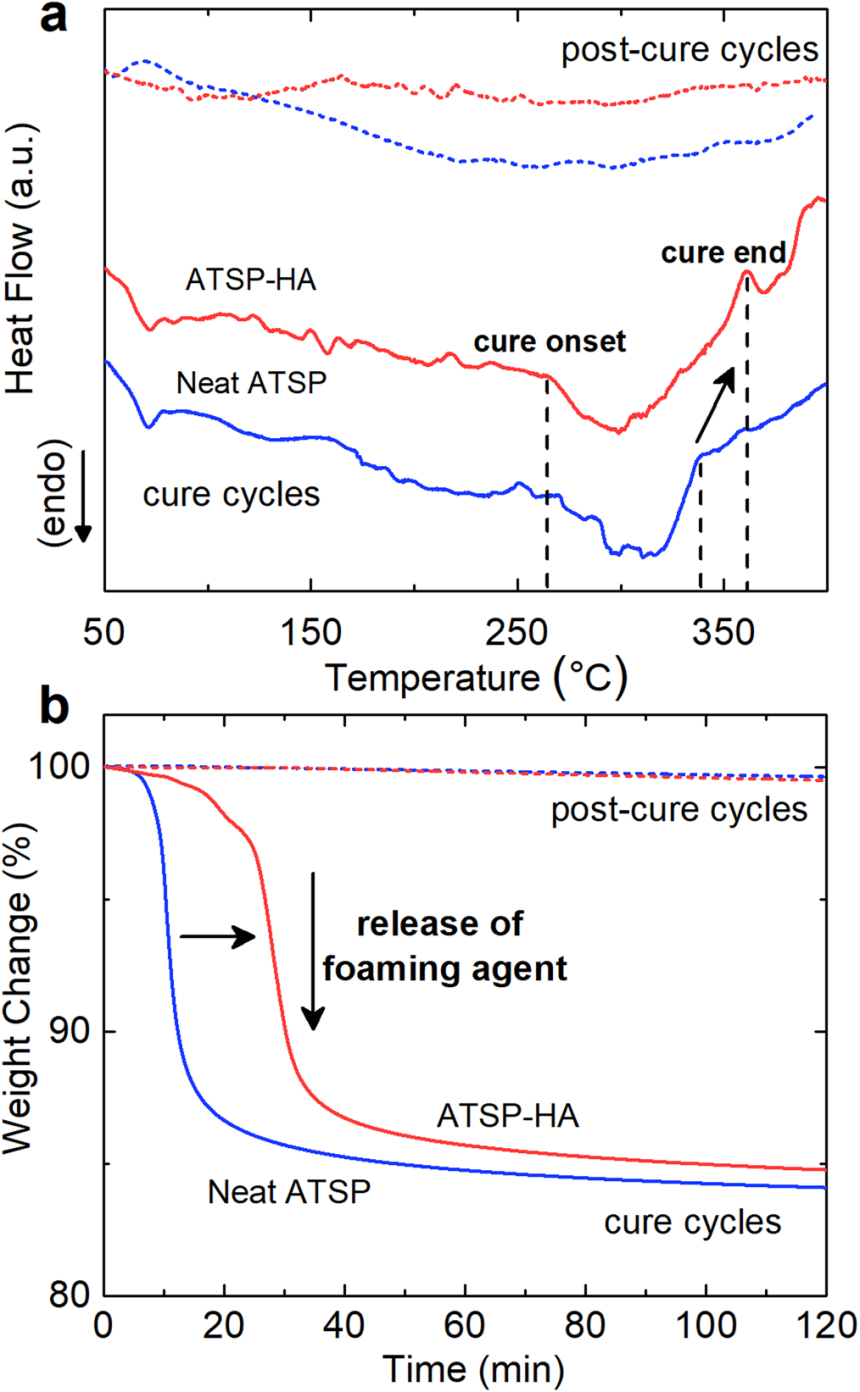
Thermal characteristics of polymerization reaction. **(a)** Differential scanning calorimetry analyses of cure and post-cure characteristics of the neat mixture of the matching oligomers (neat ATSP) and combined mixture of the precursor oligomers with hydroxyapatite particles (ATSP-HA). The DSC curves are stacked by arbitrary offsets to illustrate chemical features. **(b)** Thermogravimetric analysis characterization of cure and post-cure profiles of the neat mixture of the constituent oligomers (neat ATSP) and combined mixture of the oligomers with hydroxyapatite particles (ATSP-HA). The tests are performed under an inert atmosphere of nitrogen with a rate of 10 °C/min.

Next, we explain the influence of the HA particles on the *in situ* endothermic polymerization reaction carried out between the precursor oligomers. In this regard, a temperature-ramp Differential Scanning Calorimetry (DSC) cycle is initially applied to the combined mixture of the matching oligomers and HAs (ATSP-HA), as illustrated in Fig. 2a. In the beginning, the thermal profile shows a dimple around 70 °C which corresponds to physical softening of the oligomers^21^. Afterward, the heat flux curve maintains an endothermic behavior in which both melting and condensation polymerization reaction stages progress ordinarily. The corresponding portions of the thermal curve do not differentiate from the neat mixture of the matching oligomers (neat ATSP)^21,23^. Afterwards, the curing process takes place (denoted as cure onset) at around 260 °C, similar to that of the parent form, where the thermal curve demonstrates an endothermic cure zone. The combined mixture of the ATSP-HA displays a relatively broader cure region than that of the neat precursors where the completion of the curing process progresses beyond 360 °C. Additionally, upon the curing process, a thermal relaxation peak, a modest bulge (denoted as cure end) is observed. Following the cure cycle, subsequent post-cure analysis exhibits an isothermal characteristic curve, without any features arising from the curing process, indicating sufficient curing for the nanocomposite system.

To supplement the DSC characterization, we carry out thermogravimetric analysis of the polycondensation reaction on the curing ATSP-HA combination using a Thermogravimetric Analyzer (TGA). As noted above, the polymerization process gives off acetic acid as a reaction by-product, so the TGA characterization is sought to resolve temperature and mass changes for the acetic acid release step during the reaction. Hence, a two-stage thermal cycle is applied in which a temperature-ramp is initially performed until 330 °C, and then a temperature-hold at 330 °C is applied for 90 min^23^. Figure 2b demonstrates cure and post-cure cycles obtained from the combined mixture of the precursor oligomers and HAs (ATSP-HA). The TGA curve displays a substantial mass loss of ~15 wt% that is associated with the release of acetic acid. More importantly, the TGA analysis reveals a delayed higher temperature release of the acetic acid for the ATSP-HA combination in comparison to the neat mixture of the precursor oligomers. The subsequent gradual decline in the thermogravimetric curve within the temperature-hold region (40–120 min) corresponds to crosslinking network formation during which only small weight loss occurs, which takes place due to thermal degradation of reactive functional groups at elevated temperatures. Afterward, post-cure cycles demonstrate nearly-flat thermogravimetric curves that indicate the fully cured conditions during the first cycles for the given period.

### Characterization of nanocomposite morphology

**Figure 3:**
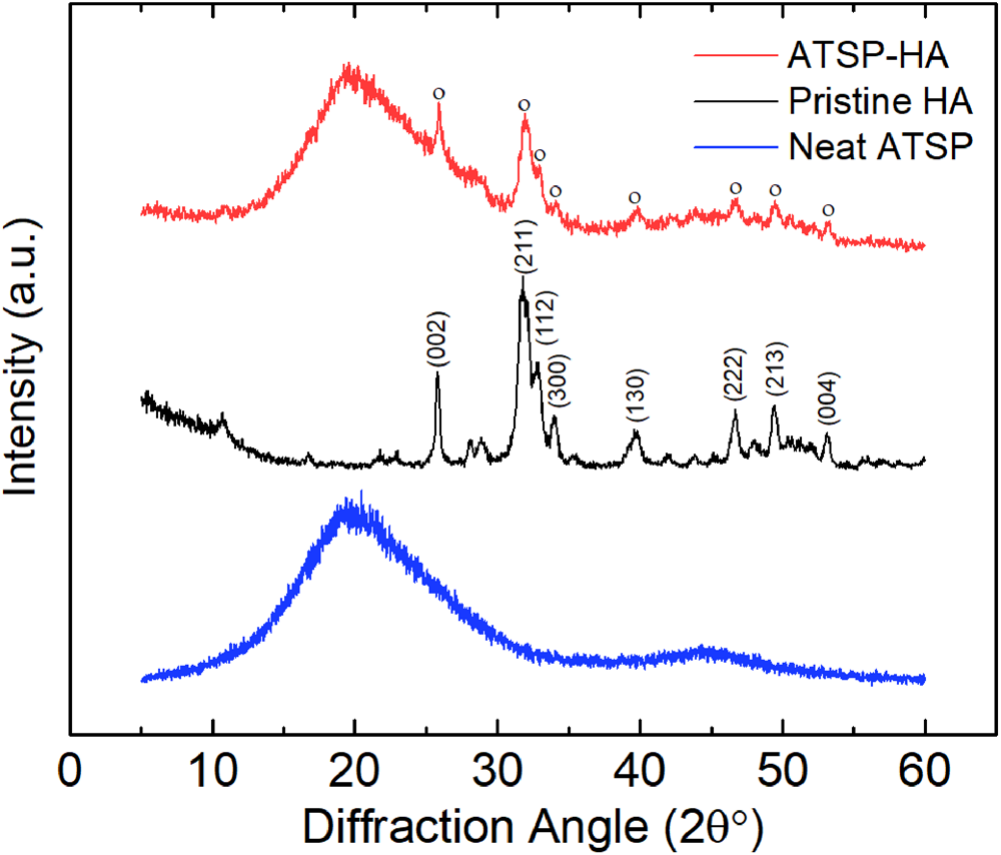
Morphological characterization of nanocomposite. X-ray diffraction characterization of neat ATSP foam (neat ATSP), pristine hydroxyapatite particles (pristine HA), and particles incorporated ATSP nanocomposite foam (ATSP-HA). The XRD curves are stacked by arbitrary offsets to illustrate morphological features.

To evaluate the structural state of the HAs within the ATSP matrix as well as investigating the overall morphology of the nanocomposite structure, we perform X-ray Diffraction (XRD) analysis. Figure 3 shows XRD spectra of the neat ATSP, pristine HA particles, and ATSP-HA nanocomposite. The neat ATSP matrix displays a very broad primary peak centered around 2θ = 20° meaning an extensively amorphous morphology^23^. Also, the pristine HAs reveal a characteristic crystalline domain for which the detected main peaks are marked in the figure. The peaks are located at 2θ = 25.8° corresponding to lattice planes of (002), 2θ = 31.8° of (211), 2θ = 32.8° of (112), 2θ = 34° of (300), 2θ = 39.7° of (130), 2θ = 46.7° of (222), 2θ = 49.4° of (213), 2θ = 53.1° of (004)^26,27^. In this regard, the ATSP-HA nanocomposite displays a similarly broad primary peak at a similar diffraction angle as the neat ATSP, which indicates that the amorphous nature of the host ATSP matrix likewise is preserved. We also observe that the characteristic peaks arising from the HAs are effectively transmitted into the spectra highlighting that the HA particles are well incorporated into the nanocomposite morphology without any physical deformation.

**Figure 4:**
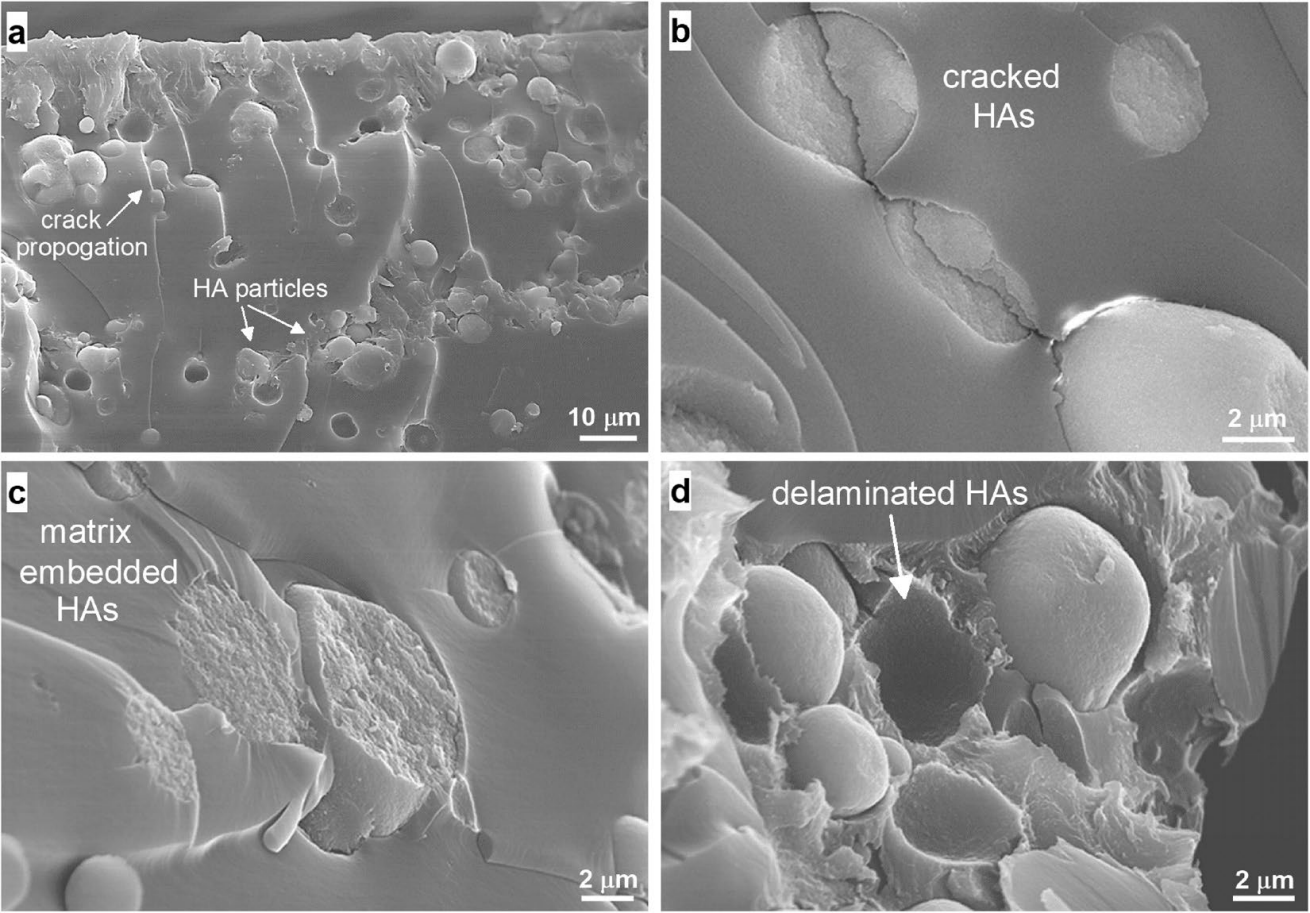
Microstructural characterization of the ATSP-HA nanocomposite. Scanning electron microscopy images obtained on fracture surfaces of the nanocomposites. Well-dispersed HAs behave as crack-arresters within the matrix **(a)**, fracture propagates through the HA particles (**b**), HAs remain broken and embedded in the matrix upon fracture **(c)**, and some HAs slip due to limited strength of interfacial coupling **(d)**.

Microstructural analysis of fracture surfaces via SEM shows several visual characteristic features of such interfacial interactions coming into effect between the HAs and ATSP matrix, as demonstrated in Fig. 4. First, the HAs are well dispersed into the matrix with minimal aggregation sites (Fig. 4a). As noticed, fracture propagates through HA particle sites. In some cases, fracture propagation results in such HA particles that crack in place, which is also a strong indication of the intrinsic physical response of the HAs being effectively transmitted to the overall physical response of the nanocomposite structure (Fig. 4b). Also, as fracture progresses and damages the HA particles, some HAs are broken and remain embedded in the matrix, which indicates the presence of an interfacial coupling between the HAs and ATSP matrix chains (Fig. 4c). We also observe that some HAs slip off the surface, so the partially reduced interfacial bonding strength is a downside of this attachment mechanism (Fig. 4d). Note that the HAs are not effectively coated with the ATSP resin such that the particles and the precursor oligomers may not effectively form *in situ* interactions during the polymerization process^23^. We also highlight that the qualitatively homogenous dispersion state, as promoted in the prepolymerization solid-state mixing stage, is effectively preserved during the polymerization reaction. Hence, to further interrogate the bonding mechanism between the HAs and ATSP backbone chains, we employ mechanical, thermomechanical and chemical analyses, as presented in the remaining text.

### Physical properties of nanocomposite structure

**Figure 5:**
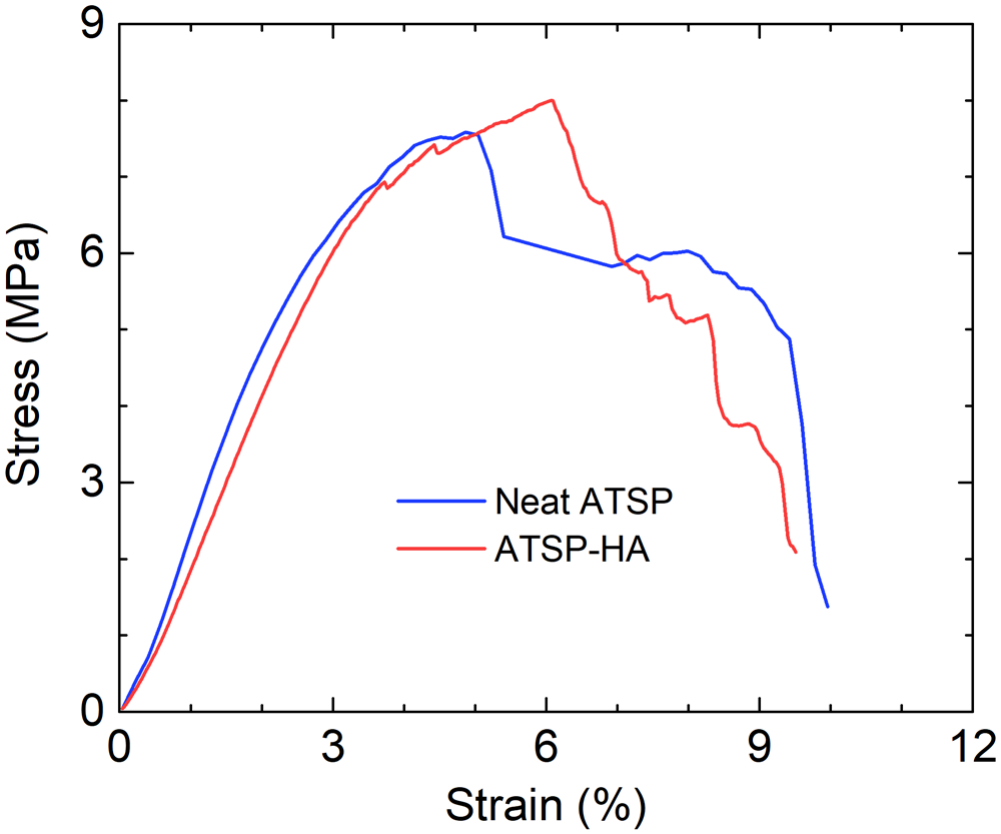
Characterization of mechanical performance. Representative compressive stress-strain curves of the neat ATSP and ATSP-HA nanocomposite foams.

To observe the extent of the crack-arresting mechanism as well as the interfacial coupling scheme on the deformation response, we perform compressive mechanical tests on the nanocomposite structure. Figure 5 shows representative stress-strain curves obtained on the neat ATSP and ATSP-HA porous nanocomposite structures. The stress-strain profiles represent the averaged results of four independent measurements taken on each sample set. The HA nanocomposite displays the two unique features of improved stress and strain at fracture. The compressive mechanical strength is increased by about 10% compared to the parent ATSP form. Also, the strain at maximum stress is enhanced by approximately 18% in comparison to the neat structure. Besides, the ATSP-HA nanocomposites become relatively softer structures where Young’s modulus decreases by approximately 15%. The densities of the two forms are almost the same (Table 1). The theoretical density of hydroxyapatite is 3.16 Mg/m^3^, which is subject to change for different sintering temperatures employed during the manufacturing process (the neat porous ATSP morphology possesses a density of 0.54 ± 0.03 Mg/m^3^)^28^.

**Table 1:** Compressive mechanical properties of the neat ATSP and ATSP-HA nanocomposite foams.

	Young’s Modulus (GPa)	Compressive Strength (MPa)	Density (Mg/m^3^)	Relative Density	Strain-to-Failure (%)
ATSP	0.27 ± 0.04	7.62 ± 0.5	0.54 ± 0.03	0.42 ± 0.03	5.34 ± 1.10
ATSP-HA	0.23 ± 0.04	8.31 ± 0.3	0.55 ± 0.01	0.43 ± 0.01	6.29 ± 0.70

The results are averaged over four specimens.

**Figure 6:**
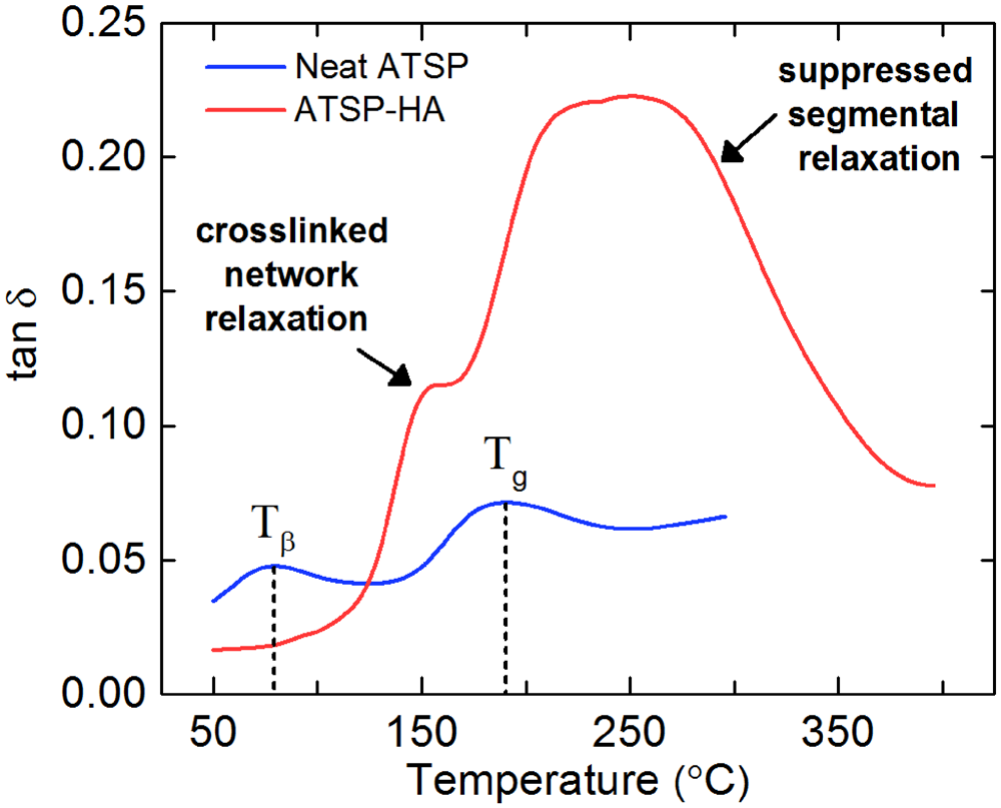
Characterization of polymer chain relaxation dynamics. Dynamic mechanical analysis tangent delta profiles of the glass transition characteristics of the neat ATSP and ATSP-HA nanocomposite .

We also analyze the glass transition behavior of the ATSP-HA nanocomposite to investigate the interfacial interactions of the HA particles and ATSP backbone chain using a Dynamic Mechanical Analyzer (DMA). The glass transition temperature is an exclusive feature of temperature-dependent structural relaxation characteristics of backbone chains for amorphous polymers, which hence can display chemical modifications induced in polymer network through nanofillers^29–31^. In Fig. 6, neat ATSP parent material exhibits two characteristic peaks of sub-glass transition (β-transition) (T_β_) at 79 °C and glass transition (T_g_) at 191 °C^21^. In comparison to the base form, the nanocomposite exhibits two distinct peaks within the glass transition domain. A low-temperature peak arises from the structural relaxation of the matrix (the crosslinked network), which, in fact, marginally downshifts to ~158 °C for the reference base material. As well, a high-temperature peak forms due to suppressed segmental relaxation of the chains incurred through the presence of the HAs (refer to the Discussion section for detailed analysis of the glass transition characteristics). In the following part, we present results of chemical analyses on the nanocomposites to unveil the nature of the interfacial attachment scheme between the HAs and ATSP.

### Interfacial interaction mechanism between ATSP matrix and HA particles

**Figure 7:**
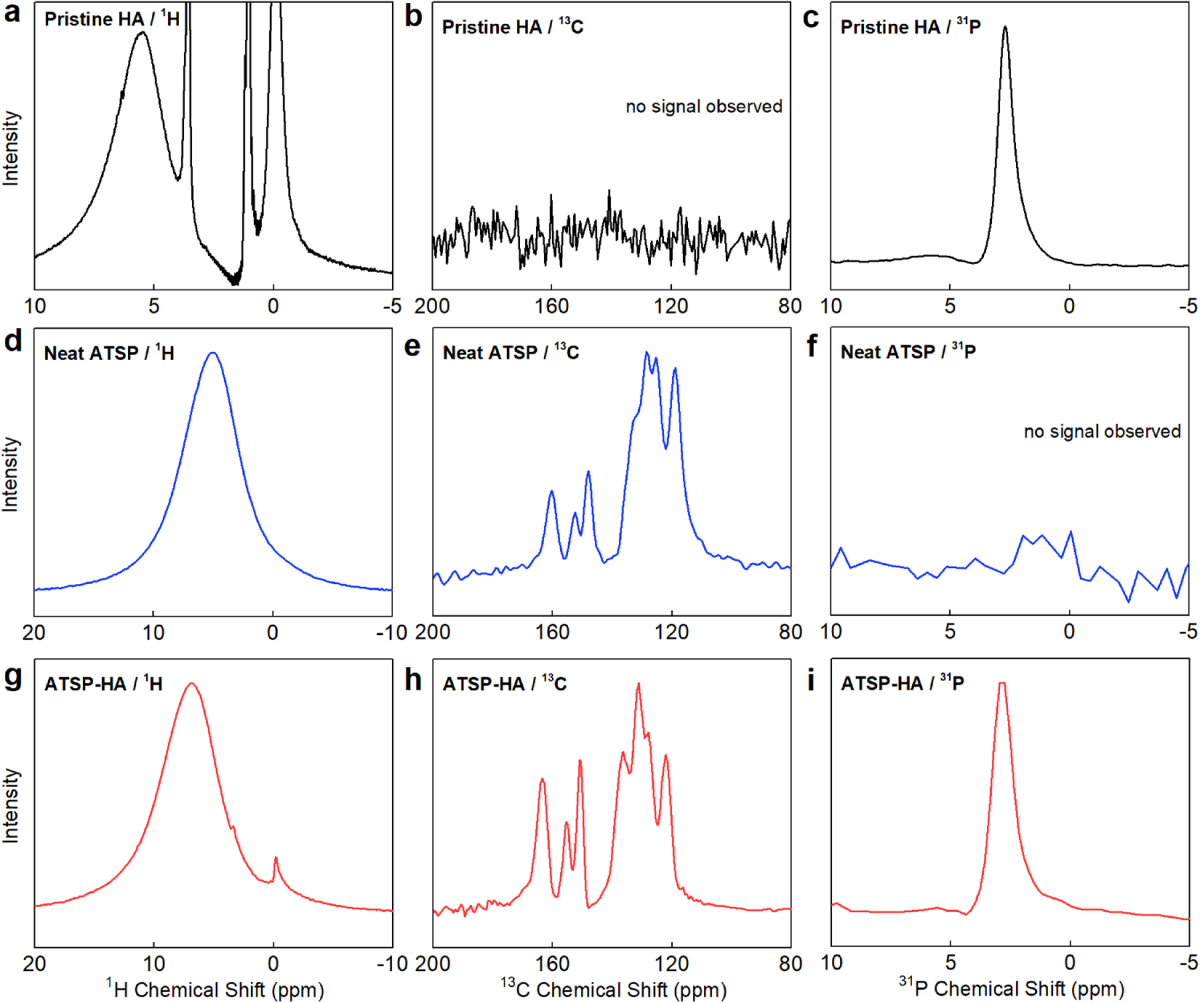
Characterization of interfacial interactions via Solid-state Nuclear Magnetic Resonance spectroscopy. Solid-state Nuclear Magnetic Resonance ^1^H direct pulse magic-angle spinning, ^13^C cross-polarization magic-angle spinning, ^31^P CPMAS spectra of the pristine HA particles (**a**–**c**), neat ATSP (**d**–**f**), and ATSP-HA nanocomposite (**g**–**i**) morphologies. No characteristic signals are observed in spectral windows of b and f. ^1^H NMR spectra were processed using 1 Hz line broadening. ^13^C and ^31^P NMR spectra were processed using 25 Hz line broadenings.

Solid-state Nuclear Magnetic Resonance (ssNMR) characterization is carried out to analyze the interfacial interactions between the HA particles and ATSP matrix. Figure 7 shows ^1^H direct pulse magic-angle spinning (DPMAS), ^13^C cross-polarization magic-angle spinning (CPMAS), and ^31^P CPMAS spectra of the pristine HA, neat ATSP, and ATSP-HA nanocomposite formulations. First, the ^1^H spectrum of the pristine HA demonstrates four peaks which are detected at −0.1 ppm, 1.0 ppm, 3.5 ppm, and 5.5 ppm (Fig. 7a). In particular, the peak at −0.1 ppm corresponds to hydroxyl ions (OH^−^), and 5.5 ppm corresponds to absorbed water H_2_O molecules^32^. The other sharp peaks (1.0 ppm and 3.5 ppm) may define apatitic functional groups in the HA structure. The neat ATSP shows a broad peak at 5.0 ppm with a linewidth of ~1640 Hz (Fig. 7d). The ATSP-HA nanocomposite exhibits a broad peak at 6.9 ppm with a linewidth of ~1844 Hz, which similarly reflects the matrix characteristics with a notable line broadening (Fig. 7g). Also, we detect two small peaks at chemical shifts of −0.1 ppm and 3.5 ppm which are likely to come from the pristine HA. Second, the ^13^C spectrum of the neat ATSP shows two distinctive peak groups corresponding to the aromatic backbone chains (C-C/C-H bonds) and the functional side-chains (C-O and C=O bonds) with a measured linewidth of ~860 Hz (Fig. 7e). For the nanocomposite, the linewidth increases to ~1428 Hz (over the highest intensity peak ~130 ppm) while preserving the original chemical configuration (Fig. 7h). Third, the ^31^P spectrum of the pristine HA displays a characteristic peak at 2.7 ppm having a linewidth of ~95 Hz, as well a small broad secondary peak is identified around 5.5 ppm (Fig. 7c). In comparison, the nanocomposite displays a peak of 2.9 ppm with a linewidth of ~125 Hz (Fig. 7i). We also perform ^31^P anisotropy measurements using a higher-field ssNMR spectrometer (data not shown) and no significant difference is observed between the pristine HA and ATSP-HA composite^33,34^. As noticed, all the elemental spectra validate a significant peak broadening through the interactions with the HAs giving rise to through ultimately altered structural relaxation behavior under a magnetic field of the backbone chains in the nanocomposite structure^35,36^. Particularly, such a peak broadening may be attributed to the interfacial entanglement of the polymer chains and the nanofiller particles causing electron mobility difference between highly crystalline HA particles amorphous ATSP chains.

## Discussion

The prime feature of the solid-state mixing approach is to characteristically establish geometrical conformity between the HAs of ~5 μm and the oligomer particles of ~50–100 μm. Such an interparticle proportionality imminently develops attractive short-range forces between the HAs and precursor oligomer particles while subsiding the van der Waals forces that are inherently present within the pristine HA particle supply^23^. Hence, the *in situ* formed attractive forces draw the HAs and ATSP oligomer particles together during the pre-polymerization process which effectively breaks the primitive HA clusters apart enabling proper distribution of the HAs within the powder oligomer domain. Furthermore, in the course of the polymerization reaction, hydrodynamic forces come into effect at elevated temperatures in molten oligomer medium enabling the acetic acid blowing agent to advance in the viscous phase. Such movement of the acetic acid bubbles facilitates rearrangement of the HAs prior to the curing and ultimately contributes to nearly homogeneous distribution in the nanocomposite morphology^21,23,37^. Also, note that the HAs preserved their original spherical morphologies following the mixing process that indicates the “nanofiller-friendly” nature of the fabrication process^23^.

Regarding the qualitative cure characterization of the precursor oligomers with the HAs, the broadening of the cure region observed in the DSC analysis may take place due to the increased molten domain viscosity of the precursor oligomers caused by the HAs. The increase in the viscosity consequently requires more energy to continue the crosslinking process, which thus stimulates premature gelation of the crosslinked matrix^38,39^. Hence, thermal relaxation peak forms due to devitrification of the aromatic matrix during the heating process in which the glass transition temperature increases beyond the cure temperature^40^. Such a thermal response indicates that the degree of crosslinking is likewise considerably affected by the presence of the HAs, as also discussed in the glass transition characteristics part. Similarly, in the thermogravimetric response of the polymerization process, the delayed release of the acetic acid indicates significant modifications in the polymerization reaction kinetics. The TGA results also concur with the outcomes of the DSC analysis that completion of the curing extends beyond the nominal temperature.

Improvements in physical properties of the bionanocomposites are directly regulated by both the physical presence and interfacial coupling of the reinforcement particles and host matrix. As observed in the SEM images, the HAs behave as crack arresters within the polymer domain which impedes further fracture propagations. Eventually, such a HA particle-conjugated network yields a more deformation tolerant morphology with increased material toughness. In correlation with the SEM analysis, several conclusions can be drawn from the compressive mechanical characterization results. In agreement with the conventional polymer nanocomposites, the ATSP-HA nanocomposite maintains simultaneous increases in the fracture strength and strain, meaning improved material toughness that would be critical advantage in the development of the artificial bone replacement nanocomposite materials with superior performance promise^41,42^. The nearly-preserved density of the structure, yet rather enhanced mechanical performance, also indicate that HAs are very well-incorporated into the matrix which develops an effective load transfer mechanism demonstrating significant improvements in the deformation tolerance. Besides, earlier studies have reported increased Young’s modulus for polymer nanocomposites with the incorporation of HAs, as also being conceivable due to significantly higher density and hardness values associated with the HAs^43,44^. However, the moderate decrease of Young’s modulus in the ATSP-HA nanocomposites may be related to modifications induced either in the cellular morphology (e.g., porosity) or the chemical structure (e.g., crosslinked polymer chain configuration) that eventually lead to considerably softer nanocomposite structures, which are highlighted in the following chemical characterization part of the text. Hence, the mechanical property improvements observed in the ATSP bionanocomposites are effectively controlled by the interfacial attachment scheme developed with the HAs by which the performance of the nanocomposite structure effectively surpasses a mere reinforcement mechanism attainable with matrix-trapped particles^45^. The results of the mechanical characterization for the improved material toughness also agree with the observations reported for the crack-arresting mechanism in the SEM microstructural analysis section.

According to the characterization of the glass transition temperature, particularly, for the low-temperature peak, in agreement with the characteristic features observed in the cure analyses, the presence of the HAs modifies the advancing crosslinking process during the polymerization process which, in this case, may promote longer polymer chains. The longer the polymer chains within the crosslinked network, the more degrees of freedom is introduced to the chains which then develops a lower temperature glass transition peak^21^. Alternatively, the free volume occupied by the HAs may induce a lower glass transition temperature for the overall matrix^46^. In this case, the interfacial attachment between the HAs and the ATSP matrix is destroyed at elevated temperatures creating free volumes in the crosslinked network that gives rise to a lower glass transition relaxation. Regarding the formation of the second peak, the physical presence of the HAs forms a pseudo-rheological percolation behavior within the matrix facilitated through short inter-nanoparticle distances, which restricts the mobility of the chains, and subsequently increases the total chain relaxation time^29,30^. The pseudo-rheological percolation is understood to be the chain confinement effect which comes into effect within a crosslinked thermoset network having strong interfacial coupling with constituent nanofiller particles. Overall, the double glass transition peak formation as a thermomechanical response of the nanocomposite structure also agrees with results shown in prior glass transition temperature literature of the nanocomposite structures^47–50^.

The interfacial bonding related line broadening observed in NMR may occur via *in situ* formed hydrogen-advanced bonds. Note that only ^1^H ssNMR spectra of the nanocomposite backbone displayed additional peaks emerging from the chemical configuration of the HAs. The precursor oligomers possess carboxylic acid functional groups which can participate in hydrogen bonding with the constituent elements in the HA structure. The hydrogen-bonding groups, via an interfacial attachment mechanism, are only operative until about 100 °C which in fact causes a lower glass transition temperature, as observed in DMA analysis^31^. Due to bond scissions at elevated temperatures near glass transition region free volumes in the chain configuration are generated which then effectively downshifts the glass transition temperature. Yet, the H-bonding mechanism is workable at room temperature, which in fact improves the mechanical performance of the nanocomposite participating in the crack-arresting deformation mechanism.

## Conclusions

We demonstrate the hydroxyapatite (HA) particle reinforced aromatic thermosetting copolyester (ATSP) matrix bionanocomposite as a potential artificial bone replacement material. The solid-state mixing based fabrication route enables near homogenous dispersion of the HAs within the ATSP matrix owing to geometrical conformity established between the HAs and precursor oligomer particles which effectively minimizes the imminent interparticle van der Waals forces. The physical presence of the HAs significantly modifies the *in situ* endothermic polycondensation reaction characteristics that consequently alters the chemical backbone configuration of the host matrix within the nanocomposite structure, as characterized using the DSC and TGA. During the polymerization reaction, the matching oligomers form a robust crosslinked network of aromatic backbone having HAs well distributed in the polymer domain, as validated through the XRD morphological analysis of the nanocomposite structure. The HAs offer a crack-arresting mechanism which develops an active load transfer network within the nanocomposite material that results in enhanced material toughness, which constitutes a critical performance factor for the artificial bone replacement applications. The glass transition characteristics of the bionanocomposite matrix are modified through nanofiller-immobilized local chain relaxation effects that give rise to double-glass transition peak formation. The ssNMR analysis on the backbone chain of the nanocomposite exhibit the presence of a hydrogen bonding-advanced interfacial coupling between the HAs and ATSP matrix through *in situ* formed hydrogen bonds demonstrating line broadening effect in the characteristic relaxation peaks. Future work focuses on the systematical biocompatibility analysis of the ATSP matrix and ATSP bionanocomposites. Also, this study may initiate further analyses of intermolecular interactions between the bioceramic particles and biocompatible polymer systems towards the improved physical performance of advanced synthetic bone bionanocomposites.

## Materials and Methods

### Fabrication of aromatic thermosetting copolyester bionanocomposite

ATSP-HA bionanocomposite is fabricated via polycondensation reaction between carboxylic acid and acetoxy functional group precursor oligomers, which generate a cross-linked aromatic polyester backbone and emit acetic acid in gas form as a reaction by-product^21^. The matching oligomers are obtained using biphenol diacetate (BPDA), 4-acetoxybenzoic acid (ABA), isophthalic acid (IPA), and trimesic acid (TMA) (Sigma-Aldrich Co., USA) at particular molar feed ratios of 1:2:3:2 and 1:0:3:3 of TMA:IPA:ABA:BPDA for the carboxylic acid-capped and acetoxy-capped oligomers, respectively. Further details of the oligomer synthesis protocols are explained in earlier works^18,20^. For the preparation of the nanocomposite, the carboxylic acid and acetoxy-capped oligomer powders (pre-mixed at 1:1 weight ratio) are combined in solid state with 10 wt% of HA particle powder (particle size: 5 μm ± 1 μm, surface area ≥100 m^2^/g) (Sigma-Aldrich Co., USA)^23^. The nanocomposite specimens are produced using a thermal cure cycle applied to the powder mixture, which includes two dwell stages at 202 °C for 90 minutes and 270 °C for 150 minutes, and a final cure stage at 330 °C for 90 minutes^21^.

### Physical characterization of aromatic thermosetting copolyester bionanocomposite

The cure characteristics of the ATSP-HA nanocomposite are analyzed using a Differential Scanning Calorimeter (DSC) (DSC 2910, TA Instruments, USA) and a Thermogravimetric Analyzer (TGA) (TGA 2950, TA Instruments, USA). The cure characteristics of the neat mixture of the matching oligomers shown in the text are taken from reference^21^. The measurements are carried out under an inert atmosphere of nitrogen. The DSC and TGA samples weigh about 20 mg. A constant 10 °C/min of heating rate is applied during both cure and post-cure cycles in the DSC and TGA analyses. The DSC thermal cycle includes only a temperature-ramp process while the TGA thermal cycle comprises a temperature-ramp stage and a temperature-hold stage for 90 min at 330 °C. As the HA-oligomer mixture is cured following the thermal cycle, it is kept under nitrogen atmosphere, to minimize exposure to the oxidative environment and thermal degradation effects, until the temperatures of the heating chambers of the DSC and TGA return to room temperature^23^.

A Scanning Electron Microscope (SEM) (S-4700, Hitachi, Japan) is employed to image microstructures of uncured powder combinations, assisted with Energy Dispersive Spectroscopy (EDS) (Oxford Instruments, UK) to perform an elemental analysis. An SEM (S-4800, Hitachi, Japan) is utilized to image fracture surfaces of the nanocomposites. SEM images are obtained in high-resolution upper detector mode at 10–15 kV voltage and 5–10 μA current.

Morphologies of the ATSP nanocomposite and pristine HA particles are characterized using a powder X-ray diffractometer (XRD) (Siemens/Bruker D-5000, USA) with Cu K-alpha source operated at 45 kV and 30 mA, and 0.15148 nm wavelength with 0.5°/s nominal scanning rate between diffraction angles of 2θ = 5° and 2θ = 60°. The XRD spectrum of the neat ATSP shown in the text is taken from reference^23^.

Compressive mechanical properties of foam morphology nanocomposite are determined using a compressive load frame (4483 Load Frame, Instron Testing Systems, USA) with a constant crosshead speed of 5 mm/min. The specimens are cylindrical with a diameter of 1.27 cm and a height of 2.54 cm. Density is calculated as the ratio of measured weight to volume of the specimens. Relative density is calculated as the ratio of the measured density of the nanocomposite foam morphology to the density of neat fully dense ATSP (1.27 Mg/m^3^). The compressive mechanical properties are averaged over four test samples, and standard deviations are given by error bars, accordingly. The mechanical property results of the neat ATSP foams shown in the text are taken from reference^21^.

Glass transition characterization of foam morphology nanocomposite is performed using a Dynamic Mechanical Analyzer (DMA) (Q800 TA Instruments, USA) operated with a dual-cantilever beam (DCB) bending fixture. Three samples are tested and all exhibit overlapping thermal profiles. A temperature-ramp cycle is operated with a 3 °C/min heating rate. The specimens are in dimensions of 35 × 10 × 5 mm^3^ (length × width × thickness), and the tests are carried out in the air. The glass transition characteristic curve of the neat ATSP shown in the text is taken from reference^21^.

Solid-state nuclear magnetic resonance (ssNMR) spectroscopy measurements are carried out using ground specimens (~50 mg) packed into NMR rotors with Varian Unity Inova 300 MHz NMR spectrometer. ^1^H spectrum is obtained using direct pulse (DP) (pulse width (pw) = 2.5 μs, recycle time (d1) = 2 s) excitation. ^13^C and ^31^P spectra are obtained using cross-polarized (CP) (^13^C; pulse width (pwH) = 2 μs, recycle time (d1) = 2 s, and contact time (tHX) = 4 ms) (^31^P; pulse width (pwH) = 2 μs, recycle time (d1) = 2 s, and contact time (tHX) = 3 ms) excitations. The specimens are spun at 10 kHz. Data processing is performed using the MestreNova software.
